# A morphogram for silica‐witherite biomorphs and its application to microfossil identification in the early earth rock record

**DOI:** 10.1111/gbi.12278

**Published:** 2018-02-27

**Authors:** J. Rouillard, J.‐M. García‐Ruiz, J. Gong, M. A. van Zuilen

**Affiliations:** ^1^ Equipe Géomicrobiologie Institut de Physique du Globe de Paris, Sorbonne Paris Cité Université Paris Diderot, UMR 7154, CNRS Paris France; ^2^ Laboratorio de Estudios Cristalográficos Instituto Andaluz de Ciencias de la Tierra Consejo Superior de Investígacìones Cientificas–Universidad de Granada Granada Spain

**Keywords:** Archean, early life, self‐organization processes, silica‐witherite biomorphs

## Abstract

Archean hydrothermal environments formed a likely site for the origin and early evolution of life. These are also the settings, however, were complex abiologic structures can form. Low‐temperature serpentinization of ultramafic crust can generate alkaline, silica‐saturated fluids in which carbonate–silica crystalline aggregates with life‐like morphologies can self‐assemble. These “biomorphs” could have adsorbed hydrocarbons from Fischer–Tropsch type synthesis processes, leading to metamorphosed structures that resemble carbonaceous microfossils. Although this abiogenic process has been extensively cited in the literature and has generated important controversy, so far only one specific biomorph type with a filamentous shape has been discussed for the interpretation of Archean microfossils. It is therefore critical to precisely determine the full distribution in morphology and size of these biomorphs, and to study the range of plausible geochemical conditions under which these microstructures can form. Here, a set of witherite‐silica biomorph synthesis experiments in silica‐saturated solutions is presented, for a range of pH values (from 9 to 11.5) and barium ion concentrations (from 0.6 to 40 mmol/L BaCl_2_). Under these varying conditions, a wide range of life‐like structures is found, from fractal dendrites to complex shapes with continuous curvature. The size, spatial concentration, and morphology of the biomorphs are strongly controlled by environmental parameters, among which pH is the most important. This potentially limits the diversity of environments in which the growth of biomorphs could have occurred on Early Earth. Given the variety of the observed biomorph morphologies, our results show that the morphology of an individual microstructure is a poor criterion for biogenicity. However, biomorphs may be distinguished from actual populations of cellular microfossils by their wide, unimodal size distribution. Biomorphs grown by diffusion in silica gel can be differentiated by their continuous gradient in size, spatial density, and morphology along the direction of diffusion.

## 1 INTRODUCTION

The search for biosignatures in the Early Archean geological record is challenging, as most sedimentary formations from this time interval have been exposed to hydrothermal fluids, and have undergone at least prehnite–pumpellyite facies to lower greenschist facies metamorphism.

Under these high temperature and high pressure conditions, rocks have recrystallized and organic matter has undergone carbonization. Therefore, any original organic remnant of microbial life preserved within them has been chemically altered and has potentially been deformed. Moreover, there are many non‐biological precipitation/crystallization phenomena leading to morphologies resembling primitive fossilized micro‐organisms. Examples are exfoliated micas (Wacey, Saunders, Kong, Brasier, & Brasier, 2015), dispersed hematite crystals (Marshall, Emry, & Marshall, 2011; Pinti, Mineau, & Clement, 2009), and migrated hydrocarbons (either biogenic or abiogenic) that filled the pore space between botryoidal quartz grains (Brasier et al., 2005). Most intriguing, however, are self‐organized life‐like silica–carbonate precipitates (Garcia‐Ruiz et al., 2003). These microstructures form spontaneously in the laboratory when alkaline‐earth metals are mixed with silica‐rich alkaline solutions in the presence of CO_2_ (García‐Ruiz, 1998). They have also been synthesized by diffusion of alkaline‐earth metals through silica gels. Recently, it has been demonstrated that these structures can form in modern serpentinization‐derived alkaline spring waters (García‐Ruiz, Nakouzi, Kotopoulou, Tamborrino, & Steinbock, 2017). Once these mineral “biomorphs” are formed, the adsorption of organic molecules (either biogenic or abiogenic) on their surface and subsequent thermal alteration during metamorphism would transform them into carbonaceous microstructures that truly resemble actual microfossils (García‐Ruiz, Carnerup, Christy, Welham, & Hyde, 2002; Garcia‐Ruiz et al., 2003; Opel, Wimmer, Kellermeier, & Cölfen, 2016). For instance, it has been suggested by Garcia‐Ruiz et al. (2003) that such a process represents a potential explanation for the controversial filamentous microfossils of the 3.5 Ga old Apex Chert, Pilbara, Western Australia (Brasier et al., 2002; Schopf, Kudryavtsev, Agresti, Wdowiak, & Czaja, 2002).

During hydrothermal serpentinization of seafloor ultramafic rocks, the pH of emanating fluids is largely controlled by temperature. Above 250°C, these fluids are slightly acidic (pH < 5), but with decreasing reaction temperature, the fluids become increasingly alkaline, reaching a pH of 11 at 50°C (Macleod, McKeown, Hall, & Russell, 1994; McCollom & Bach, 2009). This increase in pH is due to low‐temperature equilibration with brucite that forms during the serpentinization process (McCollom & Bach, 2009). The temperature of hydrothermal fluids is dependent on the depth of circulation, the average geothermal gradient, and the extent of infiltration of seawater cooling the crust. As a consequence, although the Archean geothermal gradient is believed to be higher than today (Arndt & Nisbet, 2012), fluids that altered ultramafic rocks may have displayed a wide range of temperatures and pH values. Alkaline hydrothermal serpentinization of ultramafic crust—which is observed at modern low‐temperature (<250°C) vent sites such as “Lost City” in the Atlantic Ocean (Lang, Butterfield, Schulte, Kelley, & Lilley, 2010; Proskurowski et al., 2008)—likely was an important process in aquatic environments of Hadean and Early Archean age (Russell, Hall, & Martin, 2010; Shibuya, Komiya, Nakamura, Takai, & Maruyama, 2010), when komatiites covered large areas of the ocean floor, which contained more MgO than basaltic rocks today (Arndt & Nisbet, 2012).

Early in Earth history, the concentration of silica in the oceans was much higher than today (Konhauser, Jones, Reysenbach, & Renaut, 2003; Maliva, Knoll, & Simonson, 2005; Siever, 1992), which is attested by ubiquitous sedimentary chert deposits in Archean greenstone belts (Nijman, De Bruijne, & Valkering, 1998; Van den Boorn, Van Bergen, Nijman, & Vroon, 2007; Van Kranendonk & Pirajno, 2004). These cherts formed either by direct precipitation of colloidal silica from silica‐rich seawater, or by fluid‐induced silica infiltration/replacement (silicification) of preexisting sediments (Stefurak, Lowe, Zentner, & Fischer, 2014; Van den Boorn et al., 2007). In some cases, such as in the Marble Bar chert, Pilbara, Western Australia, soft deformation and remobilization features show that the chert precursor, at least at some point of its history, was a high‐viscosity gel‐like material (McLoughlin, Wilson, & Brasier, 2008; Van Kranendonk, 2006). Debris from micro‐organisms living in the upper water column would settle down on the ocean floor, and thus become effectively entombed by silica gel. Upon burial, these amorphous silica deposits (opal‐A) would transform to cherts consisting of microcrystalline quartz in which microfossil assemblages could have been preserved. In addition to these clear sedimentary cherts, there is ubiquitous evidence for hydrothermal cherts that formed when silica‐rich seawater circulated through the igneous crustal basement. Such hydrothermal effluents, upon cooling and mixing with overlying seawater, would have precipitated silica, directly within the hydrothermal duct or near its vent, thus creating chert dykes and local layered deposits (Van den Boorn et al., 2007). Barite veins are often found associated with these hydrothermal cherts (Nijman et al., 1998; Van Kranendonk, Hickman, Williams, & Nijman, 2001) indicating that significant amounts of dissolved Ba‐ions were present in the precursor hydrothermal fluids. These hydrothermal fluids also caused serpentinization of olivine within the crust, releasing H_2_ and causing the metal‐catalyzed synthesis of CH_4_ and more complex hydrocarbons by Fischer–Tropsch synthesis (McCollom & Seewald, 2006; Milesi et al., 2015). These hydrothermal environments would also have formed an important habitat for thermophilic micro‐organisms that are capable of metabolizing reduced gases, such as hydrogen or methane (chemolithoautotrophs; Ueno, Yamada, Yoshida, Maruyama, & Isozaki, 2006). Organic remnants of chemolithoautotrophs could thus be effectively entombed in hydrothermal silica, and could have been preserved as carbonaceous microfossils in Archean chert veins. Finally, chert deposits also form in subaerial hot spring environments. When silica‐rich, and often alkaline thermal fluids reach the surface, amorphous silica is precipitated due to evaporation and/or a significant drop in temperature, leading to silica sinters in which remnants of microbial life are superbly preserved (Campbell et al., 2015; Munoz‐Saez, Saltiel, Manga, Nguyen, & Gonnermann, 2016; Ruff & Farmer, 2016). Recently, it was shown that such silica sinters with remnant microbial biosignatures (stromatolites, microbial palisade structures, gas bubbles) occurred already as early as 3.5 Ga ago (Djokic, Van Kranendonk, Campbell, Walter, & Ward, 2017).

Overall, alkaline and silica‐rich conditions (predominantly subaqueous but also subaerial) were probably frequent in serpentinization‐dominated environments during the Hadean and Early Archean, forming likely settings where life appeared and evolved (Damer, 2016; Djokic et al., 2017; Holm, 1992; Holm & Andersson, 2005; Martin & Russell, 2007; Russell et al., 2010; Schulte, Blake, Hoehler, & McCollom, 2006), but also where synthesis of silica–carbonate biomorphs could have taken place (Garcia‐Ruiz et al., 2003). On a global scale, the CO_2_ partial pressure of the Early Archean atmosphere has been interpreted to be higher than today's (Kasting, 2014; Walker, 1985; Wolf & Toon, 2014), leading to significant concentrations of dissolved carbonate species in Archean seawater and mild acidification of the seawater. Three main mechanisms can then be invoked for the formation of biomorphs in the Archean: (i) The diffusion of calcium‐rich seawater into gelified silica deposits would result in the formation of monohydrocalcite or aragonite biomorphs, (ii) the diffusion of a barium‐rich hydrothermal fluid in gelified orthochemical silica deposits would result in the formation of witherite biomorphs, and (iii) the mixing between a barium‐rich hydrothermal fluid and a silica‐rich hydrothermal fluid would result in the formation of witherite biomorphs. The potential co‐occurrence of early microbial life and mineral‐based “biomorphs” in Archean environments must therefore be taken into account in early life research, and the morphological identification of carbonaceous microstructures as ancient microfossils should be carefully assessed.

García‐Ruiz, Melero‐García, and Hyde (2009) have proposed a morphogenetic model describing the growth of silica–carbonate biomorphs. These structures can be formed with barium carbonate (witherite, which is the best studied variety), strontium carbonate (strontianite), or calcium carbonate (aragonite and monohydrocalcite; Bittarello, Roberto Massaro, & Aquilano, 2010; Voinescu et al., 2007; Zhang, 2015; Zhang, Morales, & García‐Ruiz, 2017). In an alkaline solution enriched in SiO_2_, CO_3_^2−^ (derived from the diffusion of atmospheric CO_2_), and a metal cation (Ba, Ca, or Sr), the nucleation of carbonate crystals will proceed spontaneously. Their growth causes a local decrease in pH that triggers silica polymerization in the direct vicinity of the carbonate nucleii. Growth of the carbonate crystals is therefore impeded by oligomeric silica impurities, causing the main growth front of the crystals to split repeatedly at non‐crystallographic angles. During this process, single crystal symmetry is broken, leading to the formation of dendrites that expand into the entire available volume. This episode in biomorph formation is called the “fractal growth regime.”

Upon sustained fractal growth, the amount of silica coating the witherite crystals becomes eventually high enough to interrupt the crystallization of dendrites. Precipitating silica increases the local saturation index of witherite, until new nucleation events occur on the silica coating. The dendritic material is thus replaced by a sheet‐like polycrystalline material consisting of carbonate nanorods (40 nm wide, 400 nm long) interspersed in an amorphous silica matrix, a so‐called fibrillation event (García‐Ruiz et al., 2009). The orientation of the long axis of the nanorods varies continuously and smoothly with the position of the crystal in the material, a geometrical feature which has been referred to as orientation ordering (García‐Ruiz et al., 2002). This generates an overall continuous curvature in the material, characteristic of the second episode in biomorph formation, called the “curvilinear growth regime” (García‐Ruiz et al., 2009).

In this study, we synthesized silica‐witherite biomorphs both in alkaline silica‐rich solutions and in alkaline silica gels, representing potential precursors for Archean cherts. For experiments realized in alkaline silica‐enriched starting solutions, a systematic overview is presented on the variation in morphology—defined as a “morphogram”—of witherite‐silica biomorphs that form at the surface (water–air interface), over a range of pH values and barium cation concentrations. To quantify the size distribution of biomorphs, automated measurements in optical images were also performed. In solution, biomorphs tend to aggregate, lowering the quality of these measurements. To avoid this problem, the size distribution was preferentially studied for biomorphs that were grown in silica gel. In this medium, biomorphs are dispersed throughout the gel without touching each other. solution‐based experiments and gel‐based experiments can be compared because the morphogenetic mechanism underlying biomorph growth in these two media is exactly the same (Kellermeier, Cölfen, & García‐Ruiz, 2012). As it is plausible that silica gels could have formed in certain Hadean/Archean aquatic environments, the specific diffusion‐related patterns observed in gel experiments are also discussed. The results of both solution‐based and gel‐based experiments are subsequently used to define criteria for distinction between biomorphs and microfossils, and to make an assessment of the likelihood that assemblages of biomorphs are associated with, or mistaken for, true microfossils in ancient chert deposits.

## 2 METHODS

### 2.1 Computer simulations of witherite nucleation

Numeric simulations were performed to characterize the effect of our experimental parameters on the crystallization behavior of witherite. According to classical nucleation theory (DeYoreo & Vekilov, 2003; Nývlt, Söhnel, Matuchová, & Broul, 1985), the characteristic waiting time for nucleation *T* (s) decreases with the saturation index σ of the mineral (without dimension) as shown by Equation (1). The constants *A* (s) and *B* (without dimension) are positive, and for the case of witherite, σ is defined as (Ba^2+^)(CO_3_^2−^)/*K*_s_, where *K*_s_ is the solubility constant of witherite:

(1) $$T=A*\exp(B/\sigma^{2})$$

The saturation index of witherite for each individual starting solution was computed using visualminteQ (version 3.1) simulations. The partial pressure of CO_2_ was fixed at 3.8 × 10^−4^ bar, representing ambient atmospheric level. Separate simulations were made for each individual initial barium concentration ([Ba]) in our experiments. The Cl^−^ concentration was fixed at twice the initial [Ba]. To match the experimental values measured, the pH was varied between 9.3 and 11.3, with a step of 0.1 pH units between two simulations. Only the formation of witherite and amorphous silica (gel) was allowed. The activity correction method chosen was based on the theory of Debye–Hückel for non‐ideal electrolyte solutions.

### 2.2 Synthesis of biomorphs

#### 2.2.1 Synthesis of biomorphs in solution

A 15 mmol/L silica solution was prepared by diluting 1 ml of commercial water glass (Sigma‐Aldrich, 12.5 wt % of Si) in 350 ml of millipore‐filtrated water. To obtain a range of different initial pH values, this mother solution was aliquoted in small volumes, and different volumes of NaOH or HCl solutions (0.1 mol/L) were added, from 1 ml HCl to 1 ml NaOH per 10 ml of the sodium silicate solution. In total, 21 solutions with different initial pH were produced this way. Several solutions of different BaCl_2_ concentration were prepared (giving a range in [Ba] from 1.25 to 40 mmol/L) upon dissolution of solid barium chloride dihydrate in millipore‐filtrated water. The solutions were then combined in 24‐well ultra‐clear dishes to obtain 147 synthesis media with distinct initial conditions. The dishes were covered non‐hermetically by a lid, to allow the diffusion of atmospheric CO_2_ within the solution while avoiding dust deposition that would decrease the rate of evaporation. This protocol was used twice. The first time, the morphological evolution of biomorphs was specifically tracked. The second time, the pH evolution of a subset of 22 different initial solutions was measured for 24 hr by a Thermo Scientific ROSS Ultra™ semimicro pH electrode.

#### 2.2.2 Synthesis of biomorphs in a silica gel

Silica gels were prepared by neutralizing a 10 ml silica solution (~0.5 mol/L) using 3.25 ml of an HCl solution (1 mol/L). Once the gelling process was complete, a solution of BaCl_2_ (0.25, 0.5 or 1 mol/L) was added on the top of the gel and allowed to diffuse inside. The protocol is described in more detail in Melero‐García, Santisteban‐Bailón, and García‐Ruiz (2009). Under these conditions, large biomorphs appear in a few days in the gel. This protocol was used specifically for the studies of the size distribution, as it produces well‐dispersed biomorphs, so that pictures can be easily treated during image analysis.

### 2.3 Biomorph characterization

Biomorph crystallization (first appearance, growth, and morphology) at the liquid–atmosphere interface was tracked by obtaining optical microscopy pictures of each of the 147 solutions at regular time intervals for 2 days. A petrographic microscope (Leica DM 2500 P; Institut de Physique du Globe de Paris) was used in transmission mode with a 10× objective. Two multifocus long‐range microscopes were also used for separate picture acquisition at random intervals (Nikon AZ100; Instituto Andaluz de Ciencias de la Tierra, Granada, Spain).

Optical microscopy pictures were processed using imageJ software (Abramoff, Magalhaes, & Ram, 2004). After conversion to gray level, a threshold and a binarization were applied to the pictures to separate the crystallized particles from the background. The type of threshold was critical in this aspect, as some types tended to include parts of the background to the biomorphs, whereas other types tended to discard some parts of the biomorphs to the background. A “minimum” threshold was chosen, which was consistent among all pictures and correctly separated the biomorphs from their background. The particles were then detected and analyzed using ImageJ particle analyzer, giving information about the area or the shape of the particles. All data obtained this way, in every picture, for every initial condition and time step, were stored in a five‐dimension matrix (using matlab version R2009b—MATLAB and Statistics Toolbox Release 2012b; The MathWorks, Inc., Natick, MA, USA) for further use.

After the first results had been obtained, specific conditions of synthesis representing different regimes were studied again with the same protocol in 10 ml petri dishes. At different time intervals, a fraction of the structures observed floating at the surface was retrieved, rinsed (successively in 0.01 mol/L NaOH and milli‐Q water), placed on conductive tape, and gold‐coated to be observed with a Scanning Electron Microscope (Auriga FEG, IPGP). Images were taken in secondary electron mode, either with a chamber detector or with an inlens detector. The working distance was 7 mm, except for a few pictures taken with 10 or 18 mm, and the voltage was between 3 and 10 kV.

### 2.4 Size distribution studies

Pictures of biomorphs grown in gel were acquired 2 weeks after the onset of the experiment using a direct petrographic microscope (Leica DM 2500 P, IPGP) in transmission mode with a 4× objective. For every field of view, ~30 pictures were taken at regularly increasing focal depths through the gel. These pictures were used for an extended depth‐of‐field reconstruction using imageJ (EDF plugin, EPFL), which brought all structures into focus and improved ulterior picture treatment. For comparison with biological data, a culture of cyanobacteria (*Synechocystis *sp.) from the Pasteur Cyanobacteria Collection (PCC6803) was grown with standard BG‐11 media to stationary growth phase. It was subsequently imaged with an Olympus BX‐51 microscope under 100× (oil) objective with contrast enhanced using differential interference contrast (DIC) optics. For studies on sizes, pictures were treated in the same way as presented before with imageJ. When particles tended to connect, they were separated automatically with a watershed algorithm (Beucher & Lantuejoul, 1979) which detects boundaries between areas in grayscale images. The size is given as an equivalent diameter:

(2) $$\text{Equivalent diameter}=2\sqrt{\left(\frac{\text{Area}}{\pi }\right)}$$

To quantitatively compare size distributions, their widths are characterized using (average/standard deviation) ratios. These ratios were computed for biomorphs in our experiments, a biological test sample (a picture of a culture of *Synechocystis* spp.), and size distribution data found in Archean micropaleontology literature (Butterfield & Chandler, 1992; Wacey, Kilburn, Saunders, Cliff, & Brasier, 2011, Supporting Information).

## 3 RESULTS

### 3.1 Biomorph nucleation

#### 3.1.1 Numerical simulations

**Figure 1:**
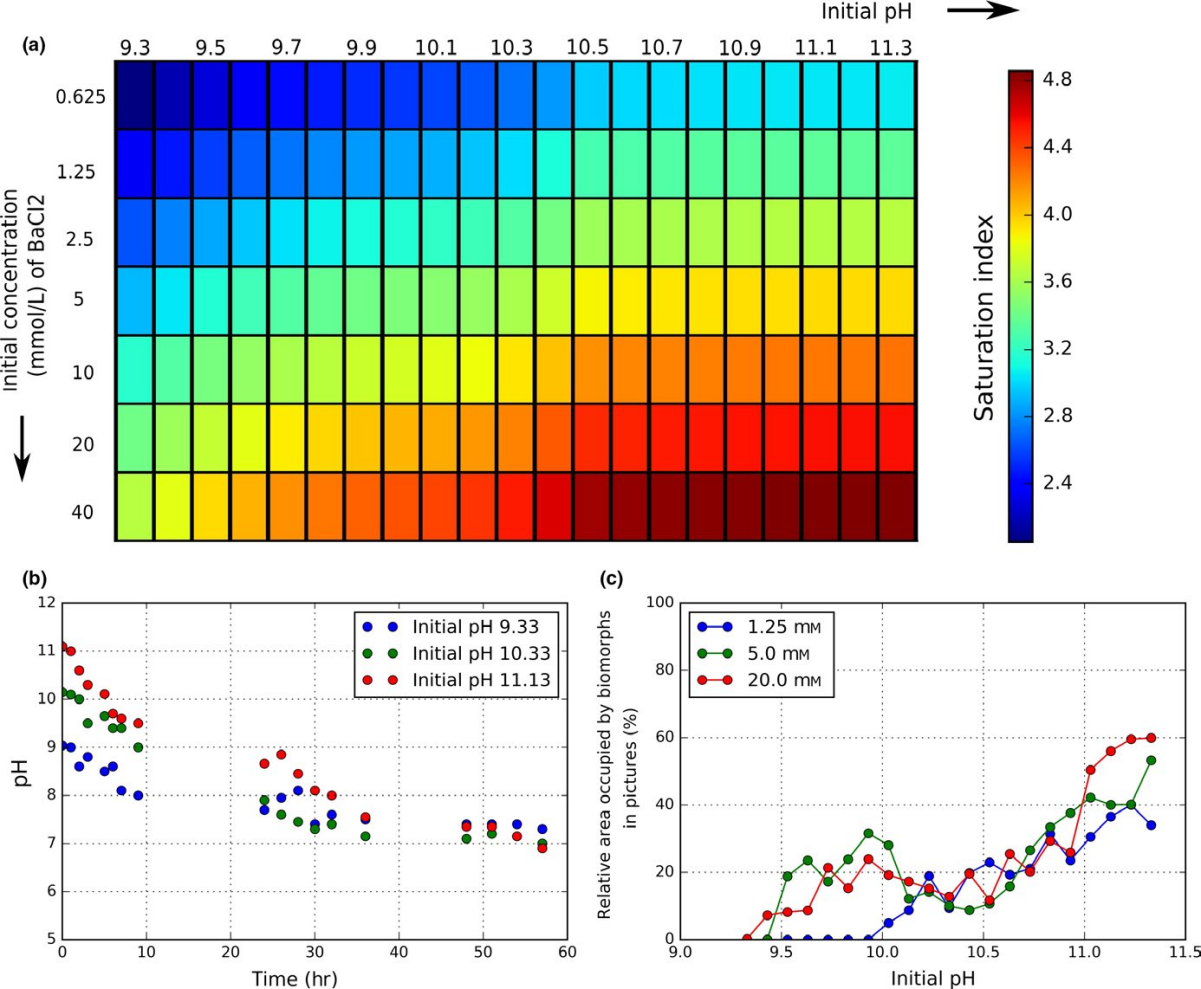
(a) Colormap showing the saturation index of witherite for the different initial settings of our solution experiments. (b) Evolution of the pH in the synthesis medium during the reaction for different initial pH values. (c) Relative area covered by biomorphs in pictures after 26 hr depending on the initial pH and for different initial [Ba] [Colour figure can be viewed at http://wileyonlinelibrary.com]

Using numerical simulations described in paragraph 3.1, for each starting solution, the saturation index σ of witherite was calculated. The results are shown in Figure 1a. Each initial condition is represented in a single cell as a function of both initial [Ba] and initial pH shown, respectively, on the vertical and the horizontal axis. As the [CO_3_^2−^] increases with the pH, the axes of this figure can also be read as initial [Ba] and initial [CO_3_^2−^]. Therefore, the saturation index of witherite, represented by a color chart, increases from left to right and from top to bottom.

#### 3.1.2 Solution experiments

Experiments of nucleation in solution were performed for each of the individual starting conditions shown in Figure 1. Within a few minutes to a few hours (depending on the initial conditions—see Table 1) after reagents had been mixed, biomorphs were observed at the water–air interface. In accordance with Equation (1), Figure 1a and Table 1, particles tend to appear earlier at higher initial [Ba] and higher initial pH (higher saturation indexes). The precipitates also developed on the plastic walls, at depths increasing with time, and eventually reached the bottom of the wells. This is due to the progressive diffusion of atmospheric CO_2_(g) from top to bottom, causing an increase in the saturation index of witherite. Due to the continuous diffusion of CO_2_(g) into the reaction medium, the pH value drops consistently after the start of the experiment, undergoing an exponential decrease and reaching neutral values in a few tens of hours (Figure 1b). Our results also illustrate that owing to the increased rate of CO_2_ uptake at higher pH values, the pH decreases faster for solutions starting at more alkaline conditions, an observation already made by Eiblmeier, Kellermeier, Rengstl, García‐Ruiz, and Kunz (2013). This results in equal equilibration times for all the solutions (around 45 hr). Although the general exponential decrease of the pH in our experiments is in accordance with previous results (Eiblmeier et al., 2013; Kellermeier, Melero‐García et al., 2012), the measured rate is two to three times higher. This can be ascribed to differences between the experimental protocols. Our experimental protocol for pH measurement involved frequent opening of the wells, thus accelerating the rate of CO_2_ uptake. At the end of the experiments, the precipitate coverage at the water–air interface (given in relative area, %), as a function of initial pH and starting [Ba] is shown on Figure 1c. Overall, the area covered by the precipitates increases with increasing initial pH, while the influence of the initial [Ba] is less conclusive. As the pH value is controlling the carbonate concentration in solution, the extent of biomorph growth is mostly controlled by carbonate availability and not by barium availability.

**Table 1:** Table summarizing the time of appearance of biomorphs (measured by optical microscopy) for different initial pH (columns) and [Ba] (lines). The different time intervals are as follows: 0.5: biomorphs have appeared in optical microscopy before 0.5 hr, 1.5: biomorphs have appeared between 0.5 and 1.5 hr, 2: betw. 1.5 and 2 hr, 3: betw. 2 and 3 hr, 4: betw. 3 and 4 hr, 5: betw. 4 and 5 hr, 6: betw. 5 and 6 hr, 8: betw. 6 and 8 hr, 23: betw. 8 and 23 hr, Nf: not found after 23 hr

	9.33	9.43	9.53	9.63	9.73	9.83	9.93	10.03	10.13	10.23
1.25 mmol/L	Nf	Nf	Nf	Nf	Nf	Nf	Nf	23	23	23
2.5 mmol/L	Nf	Nf	Nf	Nf	Nf	6	6	8	8	23
5 mmol/L	Nf	Nf	6	4	4	2	3	5	4	4
10 mmol/L	Nf	Nf	4	2	2	2	2	2	3	3
20 mmol/L	Nf	Nf	3	1.5	1.5	1.5	2	1.5	2	0.5
40 mmol/L	Nf	Nf	1.5	1.5	1.5	0.5	0.5	1.5	1.5	1.5

	10.33	10.43	10.53	10.63	10.73	10.83	10.93	11.03	11.13	11.23	11.33
1.25 mmol/L	23	23	23	3	3	3	3	3	0.5	0.5	0.5
2.5 mmol/L	4	4	3	1.5	0.5	0.5	0.5	0.5	0.5	0.5	0.5
5 mmol/L	3	3	3	0.5	0.5	0.5	0.5	0.5	0.5	0.5	0.5
10 mmol/L	3	3	3	3	1.5	0.5	0.5	0.5	0.5	0.5	0.5
20 mmol/L	1.5	3	3	2	2	0.5	0.5	0.5	0.5	0.5	0.5
40 mmol/L		3	2	3	1.5	1.5	0.5	0.5	0.5	0.5	0.5

#### 3.1.3 Gel experiments

**Figure 2:**
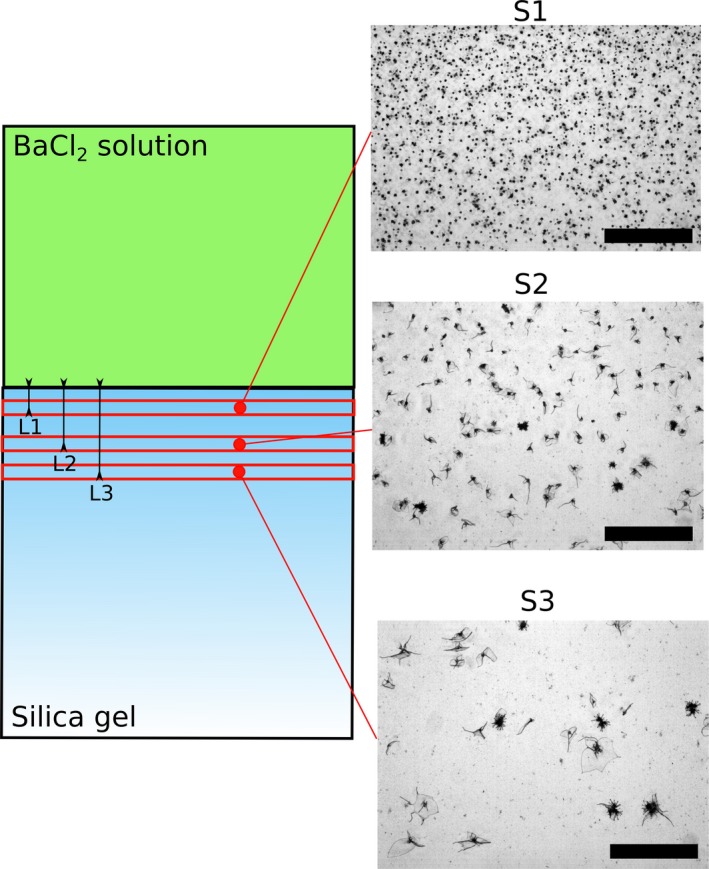
Crystallization gradient of biomorphs in a gel. S1, S2, and S3: pictures (optical microscopy) taken at different distances from the diffusion source (L1: 3 mm, L2: 8 mm, L3: 12 mm). Scalebars: 600 μm [Colour figure can be viewed at http://wileyonlinelibrary.com]

Within a carbonate‐rich silica gel, the precipitation of biomorphic witherite is caused by the diffusion of barium through the medium. The barium concentration, and therefore also the saturation index σ of witherite, decreases spatially away from the diffusion source (Melero‐García et al., 2009). Pictures taken at equal times and at different distances from the initial diffusion source show that the spatial concentration of biomorphs decreases with the distance to the diffusion source (Figure 2), which is in accordance with this decrease of σ away from the source, and with observation in previous studies (Melero‐García et al., 2009). While in solution experiments, the biomorphs tend to grow on interfaces (air–water interface, solid–water interface), they form throughout the gel under conditions which vary with time and space, thus displaying a continuous variation in morphologies.

### 3.2 Biomorph morphology

#### 3.2.1 Biomorphs in solution experiments

**Figure 3:**
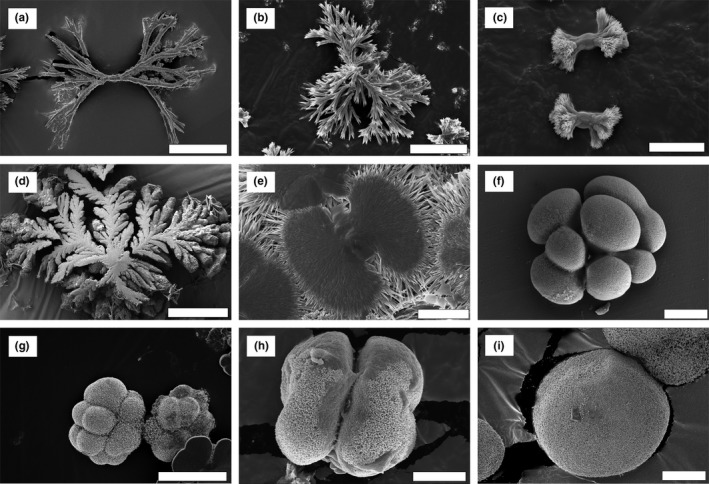
Main types of biomorphs obtained through fractal growth. (a), (b), (c) Dendrites. (d) Fern‐like biomorph. (e) to (h) Framboidal‐type biomorphs. (i) Spheroidal biomorph. Pictures are taken using Scanning Electron Microscopy (secondary electron mode). Scalebars: (a), (b) 10 μm; (c), (e), (f), (h), (i) 5 μm; (d), (g) 20 μm

**Figure 4:**
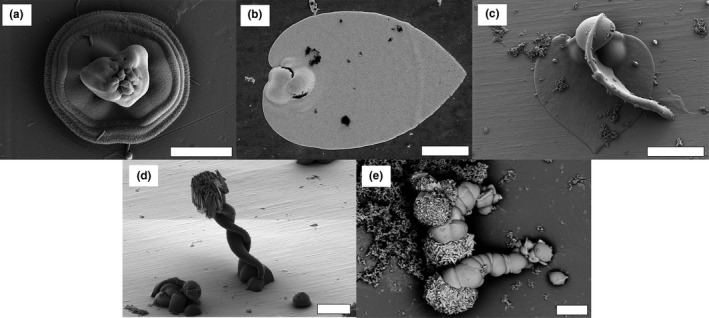
Examples of biomorph types obtained through curvilinear growth and explained by the morphogenetic model proposed in García‐Ruiz et al., 2009. (a) Discoidal sheet, with characteristic wave‐like patterns. (b, c) Leaf‐like biomorphs. (d) Helical braid. (e) Wormlike braids. Pictures are taken using Scanning Electron Microscopy (secondary electron mode except E taken in backscattered mode). Note the growth of large witherite crystals on the surface of some biomorphs, related to post‐removal drying. Scalebars: (a) 10 μm; (b), (c), (d), (e) 20 μm

**Figure 5:**
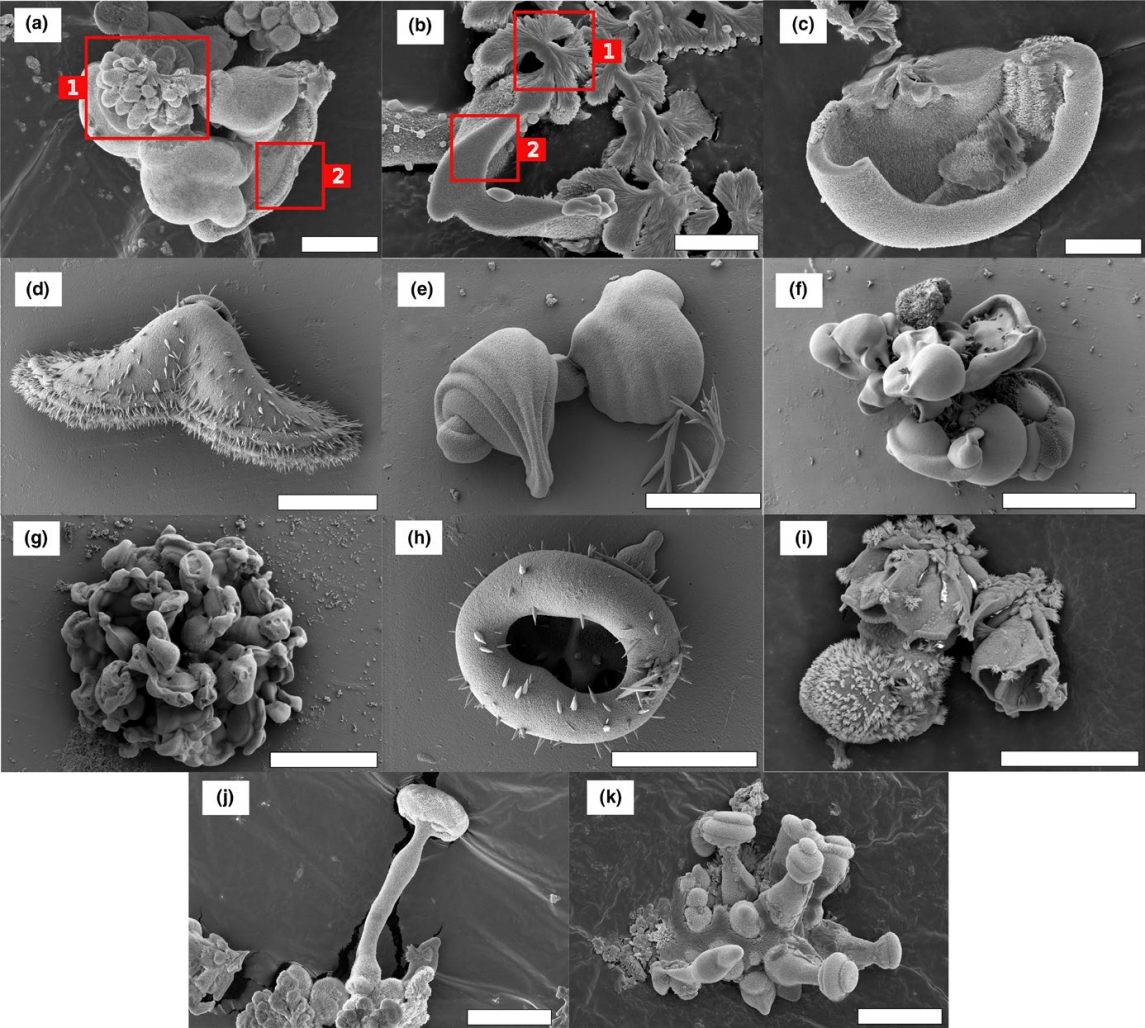
(a) and (b) transition between fractal growth and curvilinear growth. The squares noted 1 and 2 indicate respectively zones of fractal growth and of curvilinear growth in the same structure. (c–k) Other types of biomorphs obtained through curvilinear growth. (c–g) biomorphs obtained through the involution of folding sheets. (c) Gutter‐like biomorph. (d) Moth‐like biomorph. (e) Snail‐like biomorphs. (f, g) involuted aggregates. (h, i) biomorphs obtained through the development of closed sheets. (h) donut‐shaped biomorph. (i) Coral or urn‐shaped biomorphs. (j, k) mushroom‐like biomorphs. Pictures are taken using Scanning Electron Microscopy (secondary electron mode). Scalebars: (a), (d), (e), (h), (j), (k) 10 μm; (b), (c) 5 μm; (f), (g), (i) 20 μm [Colour figure can be viewed at http://wileyonlinelibrary.com]

A wide diversity of carbonate‐silica biomorph shapes were observed in the solution experiments (Figures 3, 4, 5). These shapes can be classified into several main morphological types, following the morphogenetic model presented in earlier studies (García‐Ruiz et al., 2009; see the Introduction).

The first growth regime represents fractal growth. The variation in the number of non‐crystallographic branching events along a certain length of crystal, or branching density, appears to be responsible for the variety of fractal growth shapes observed. At low branching density, the individual branches are physically separated from each other within the overall structure, leading to dendrite‐like shapes (Figure 3a,b). At higher branching density, space constraints force the branches to decrease in diameter at every splitting event (Figure 3c), and finally connect to form a smooth overall fern‐like dendritic structure (Figure 3d). Eventually, this splitting leads to a continuous growth front of witherite crystals, leading to framboidal (Figure 3e–h) and ultimately spherical shapes (Figure 3i).

The second growth regime represents curvilinear growth. One of the prominent aspects of this regime is the potential curling of sheet‐like material on its outer edge (García‐Ruiz et al., 2009). Many of the shapes formed during this regime can be understood in terms of growth front speed and curling speed, leading to simple circular sheets (no curl, growth occurs at a similar rate in every direction, Figure 4a), leaf‐shaped biomorphs (due to an encounter of two curls of opposite handedness, Figure 4b,c), and helicoids (due to an encounter of two curls of the same handedness, Figure 4d,e). If the two curls have the same propagation velocity as the radial velocity of the sheet, a perfect helicoid is formed, but if the curling velocity exceeds the radial velocity, the two rims coil on each other, creating a braid (Figure 4d). If, in addition, one of the curls propagates faster than the other, a wormlike structure is created (Figure 4e).

The transition from fractal growth to curvilinear growth can be easily observed in some structures, with a core of fractal growth aggregates, surrounded by radially expanding sheets which were formed by curvilinear growth (Figure 5a,b). Instead of twisting, the sheets can also fold onto themselves, forming gutter shapes (5), moth‐like shapes (5d), snail‐like shapes (5e), or combinations of these (5f,g). In other cases, ring‐like structures arise through the curvilinear regime and grow with diverse thickness and to diverse heights to form donut (5h), coral, or urn‐like shapes (5i). Lastly, mushroom‐like shapes (Figure 5j,k) can emerge from the fractal aggregates. Their formation process is not well understood yet.

Looking at a single experiment, at a given time, only one type of growth process occurs (fractal or curvilinear). However, the morphologies of individual biomorphs vary. For example, circular sheets can grow next to leaf‐shaped biomorphs, or spheroids can grow next to framboids.

#### 3.2.2 Biomorphs in silica gel experiments

The growth of witherite in gels occurs by a counter‐diffusion process (Melero‐García et al., 2009). A first precipitate of metal carbonate is created at the gel–solution interface at a pH close to 8.5 due to mixing of the acidic metal solution and the alkaline gel. As the acidic metal solution diffuses across the gel, it becomes less concentrated. Therefore, within a single gel experiment, there is a gradual variation in conditions across the gel, from relatively acidic and metal‐rich at the interface to more alkaline and carbonate‐rich at the end of the gel. As a consequence, in the gels prepared for this study, the shape of biomorphs changes along the diffusion direction (Figure 2). Close to the diffusion source, the low pH values lead to the appearance of biomorphs related to the fractal growth regime. This was observed before in experiments of Melero‐García et al. (2009). Further away from the diffusion source, pH values range up to 10.5, and biomorphs display sheets, trumpet‐shapes and helicoids characteristic of the curvilinear growth regime, with more numerous and longer helicoids observable at increasing distances from the diffusion source (see also figure 9 *in* Melero‐García et al., 2009).

### 3.3 Influence of pH and [Ba] on biomorph growth in solution experiments

**Figure 6:**
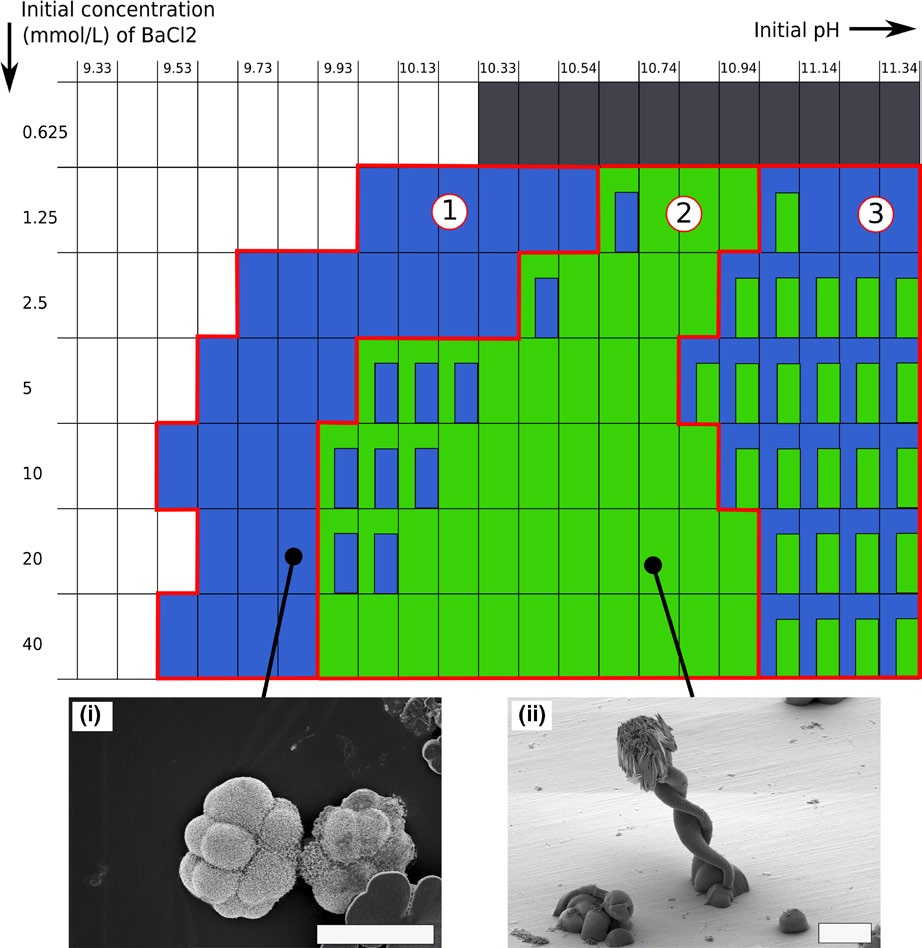
Qualitative morphogram representing the dominant growth regimes observed for each unique initial setting of pH and Ba concentration. The black shade represents the occurrence of non‐biomorphic witherite crystals. The blue shade represents the fractal growth regime. The green shade represents the curvilinear growth regime. Smaller rectangles indicate the transition to the other regime during time. The three domains outlined in red correspond to the three domains defined in the text. Scalebars: 20 μm [Colour figure can be viewed at http://wileyonlinelibrary.com]

To study the effect of solution properties on the morphological variation in biomorphs, a set of experiments was conducted in which the [Ba] was varied from 0.5 to 40 mmol/L and the starting pH was varied from 9.3 to 11.3. The experiments demonstrated a strong control of these parameters on the morphologies obtained. In Figure 6, the conditions for normal crystallographic growth of witherite crystals (black), fractal growth regime of biomorphs (blue), and curvilinear growth regime of biomorphs (green) are shown. In case of a time evolution, an overlay of a smaller rectangle indicates the secondary regime. At [Ba] = 0.625 mmol/L, at any starting pH, no biomorphs are formed. Instead, needle or prism‐like pseudohexagonal witherite crystals develop slowly at the water–air interface. At [Ba] values of 1.25 mmol/L and above, biomorphs are seen to develop, but only in the high pH range. In this high pH range, [CO_3_^2−^] is sufficient to cause the development of silica‐witherite biomorphs. When [Ba] rises from 1.25 to 5 mmol/L, the minimum pH at which biomorphic growth occurs is shifted to lower values (from 10 to 9.5), thus expanding the range of pH conditions for which the synthesis of biomorphs is possible in these experiments. In many biomorph synthesis protocols, a [Ba] of 5 mmol/L is used (Kellermeier, Glaab, Melero‐García, & García‐Ruiz, 2013; Voinescu et al., 2007). We therefore first describe the biomorphs that form at [Ba] = 5 mmol/L and variable starting pH values.

**Figure 7:**
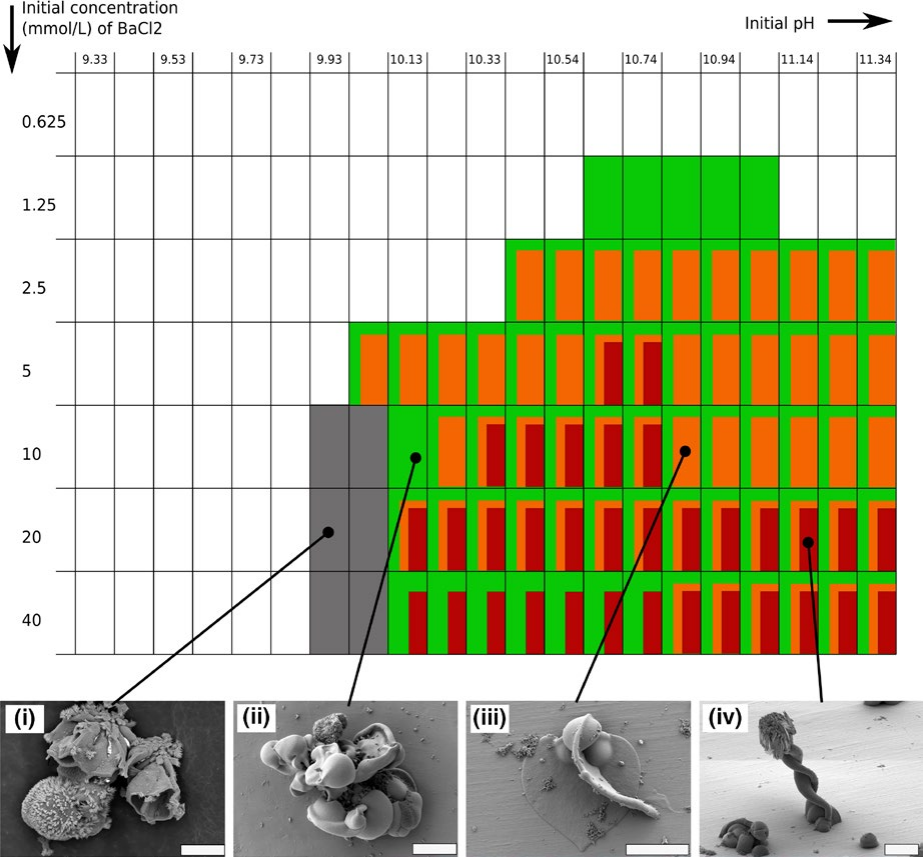
Qualitative morphogram representing the main morphologies appearing through curvilinear growth for each unique initial setting of pH and Ba concentration. The green shade represents aggregates of involuted sheets. The orange shade represents flat sheets and leaf‐like biomorphs. The brown shade represents the helical biomorphs (helicoids, braids, and wormlike braids). The gray shade represents coral‐like biomorphs. The smaller the rectangle, the later the corresponding morphology appears. Scalebars: (I), (II) 10 μm; (III), (IV) 20 μm [Colour figure can be viewed at http://wileyonlinelibrary.com]

At [Ba] = 5 mmol/L and starting pH = 9.5, fractal growth‐related biomorphs (dendritic, fern‐like, and framboidal shapes) grow for about 1 day in the medium, reaching sizes up to several hundreds of μm (Figure 6). When the starting pH is increased, between pH = 10.0–10.7, this fractal growth shifts very quickly (after a few tens of minutes) to a curvilinear growth regime. Intricate agglomerates created by involute sheets appear. After a few hours, open sheets emerge from these aggregates, leading to diverse leaf‐like and helical structures. In Figure 7, the detailed pH‐[Ba] conditions are shown at which the different curvilinear structures are formed, including involute sheets (green), leaf‐like sheets (orange), coral‐like structures (gray), and helical structures (brown). In case of a succession of morphologies, an overlay of a smaller rectangle indicates the later formed phase. As is seen in Figure 6, at pH above 10.7, the same fractal growth‐related structures appear as those that formed at pH 9.5–10.0. However, they appear much faster (within a few minutes after mixing of the reagents), and are much more numerous and smaller, around 10–15 μm in size. This is in accordance with previous results presented by (Eiblmeier et al., 2013). After a few hours, at these conditions, new biomorphs appear, with a morphogenetic path very similar to what was described previously for pH 10–10.7. Based on these observed differences, we can define three general domains for biomorph growth, separated by red lines and numbered as follows in the Figure 6: 1) a low pH regime, defined by slow fractal growth of a small number of large biomorphs, 2) An intermediate pH regime, defined by fast fractal growth, followed by curvilinear growth of involute complex aggregates, flat sheets, and helical structures, 3) A high pH regime, defined by short fractal growth of a large number of small biomorphs. Although all the morphologies could be interpreted as being a part of the same theoretical morphological continuum (Melero‐García et al., 2009), see also section 4.3), this complete continuum is not observed under all experimental conditions. In domain 1, the development of biomorphs stops at fractal growth and is not followed by a curvilinear growth. In domain 2, the fractal growth is very short, and often not visible in the final structures because covered by the subsequent folding of sheets. The pH range of these domains was modified when the [Ba] was changed. An increase in [Ba] expands the pH range of domain 2 (see Figure 6). The range in pH over which helical structures are synthesized is significantly increased as well when [Ba] increases from 5 to 40 mmol/L (see the gray area in Figure 7). They are found for pH = 10.1–11.3 (upper limit of our study) for [Ba] above 20 mmol/L. Overall, the higher the concentration of barium, the larger the extension of the curvilinear growth regime.

After a few days, in most experiments, prism‐ and needle‐like witherite crystals start covering the surfaces of previously formed biomorph shapes (e.g., Figure 5d; Kellermeier, Melero‐García et al., 2012).

### 3.4 Size distribution of biomorphs

**Figure 8:**
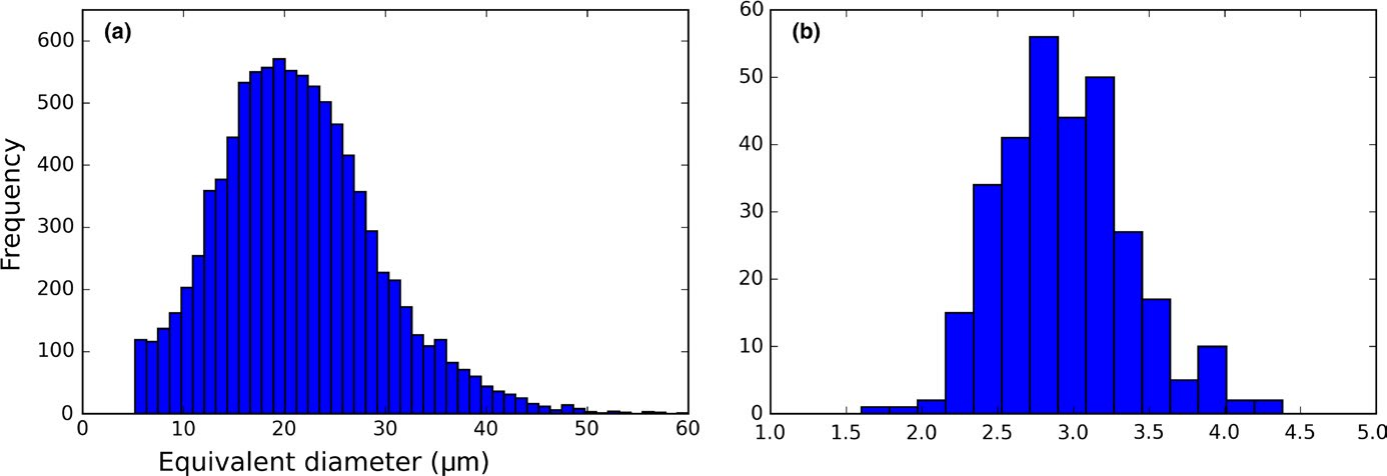
Size distribution of gel‐grown biomorphs (a) at 3 mm from the diffusion source (see 4.6) compared to the size distribution of *Synechocystis* sp. (b) [Colour figure can be viewed at http://wileyonlinelibrary.com]

In the case of solution growth, due to the physical effect of the meniscus in the well, biomorphs tend to aggregate in the center of the well, making the accurate size distribution analysis a difficult task. As a consequence, the size distribution analyses were realized in gel growth experiments. In these experiments, the slow diffusion of the barium solution leads to the formation of larger biomorphs (up to 3–4 mm) than the ones obtained in solution (up to ~200 μm for the same curvilinear growth regime). As discussed previously (section 4.1), the pH, [Ba^2+^] and nucleation index change both in time and along the direction of diffusion in the silica gel. A wide range of growth conditions thus exists in a single gel, enabling the formation of different morphologies (section 4.2). Moreover, on a mm‐scale, the number of biomorphs decreases with the distance from the diffusion point, whereas their individual size increases. This spatial gradient is illustrated in Figure 2 (S1 and S3 are separated by 9 mm). At a given distance from the diffusion point, the nucleation rate is assumed to be constant. Therefore, the size distribution of biomorphs was measured in a group of pictures aligned along a plane parallel to the diffusion front and separated from the diffusion source by 3 mm. In this area, the growth conditions are assumed to be similar, meaning that the differences in saturation index, pH or Ba/CO_3_ are not large enough to affect the growth. The obtained size distribution (Figure 8a) is unimodal, which is expected for a uniform growth process, and is close to a normal distribution, with an average size of 21 μm and a standard deviation of 8 μm. The average, standard deviation, and average/standard deviation ratios of this size distribution are summarized in the Supporting Information (Table 1) and compared to the same values in other size distributions (see section 3.4).

## 4 DISCUSSION

### 4.1 Influence of [CO_3_^2−^] diffusion on biomorph growth in solution experiments

**Figure 9:**
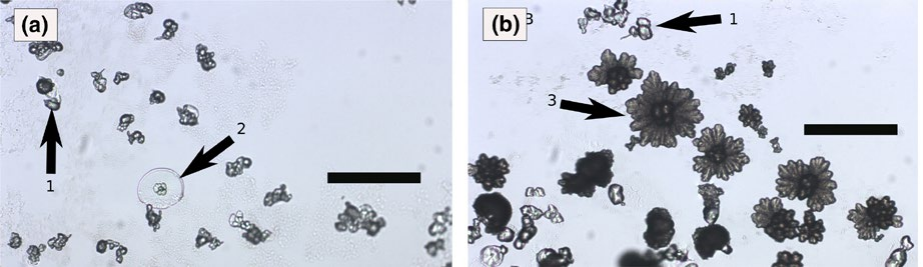
(a, b) Optical micrographs (transmission mode, scalebars 200 μm) of the particles observed at the surface of the solution for an initial Ba concentration of 1.25 mmol/L and an initial pH of 10.64. (a) picture taken after 4 hr. (b) picture taken after 26 hr. Arrow 1: Involuted sheets. Arrow 2: Flat sheets. Arrow 3: Framboidal biomorphs [Colour figure can be viewed at http://wileyonlinelibrary.com]

During the experiments, pH measurements of the bulk solution show that CO_2_ diffusion from the atmosphere progressively acidifies the growth medium (Figure 1b). We hypothesize that during this gradual change, the solution crosses the previously defined pH‐dependent crystallization domains, and generates the corresponding biomorph shapes. It is thus expected that biomorphs corresponding to more acidic domains will start growing later than those corresponding to more alkaline domains. This coexistence of different domain‐corresponding biomorphs is indeed observed in our experiments (Figure 9a,b). For the lowest initial pH values of the domain 2, involuted sheets appear first (arrows noted 1 in Figure 9), associated after 3–4 hr to flat sheets (arrow noted 2 in Figure 9). After 1 day, large framboidal biomorphs (arrow noted 3), corresponding to the domain 1, appear in the medium. It therefore appears that the synthesis medium has reached pH values characteristic of domain 1, and the growth regime has switched from a curvilinear to a fractal‐type growth process. Similarly, in the whole range of domain 3, the initial development of small framboidal biomorphs is followed by the growth of involuted aggregates and other curvilinear structures corresponding to domain 2. The synthesis medium has reached pH values characteristic of the domain 2. The final pH after equilibration with the atmosphere is the same for all the solutions (approximately at pH = 7—see Figure 1b). Therefore, solutions starting in domain 3 (the highest pH related domain) will cross the conditions related to domains 2 and 1 during their evolution. However, the coexistence of morphologies seems limited. No morphologies corresponding to domain 1 can be observed in the solutions starting in the domain 3 or in the higher pH part of the domain 2. A plausible explanation is that when the solutions reach the conditions of domain 1, the saturation index of witherite is not sufficient anymore to allow further nucleation or growth of biomorphs, and the morphologies corresponding to the domain 1 will not be formed. As was discussed in section 3.1, the rate of pH decrease is higher for higher initial pH. For the whole range in initial pH's of domain 3, the conditions related to domain 2 will be reached while the saturation index of witherite is above the limit to nucleate biomorphs corresponding to domain 2. For solutions starting in domain 2, however, the pH will decrease much slower, and the solutions starting at high pH of this domain will only reach the conditions of domain 1 when the saturation index is under the limit allowing growth of domain 1‐related biomorphs. The shapes of the observed biomorphs, their evolution throughout the duration of the experiment, and their association with other biomorph shapes can all be related to crossing by the solution of various growth‐controlling pH and [Ba] domains.

### 4.2 Morphology as a criterion to detect biomorphs in the fossil record

#### 4.2.1 Morphology of solution‐grown biomorphs

**Figure 10:**
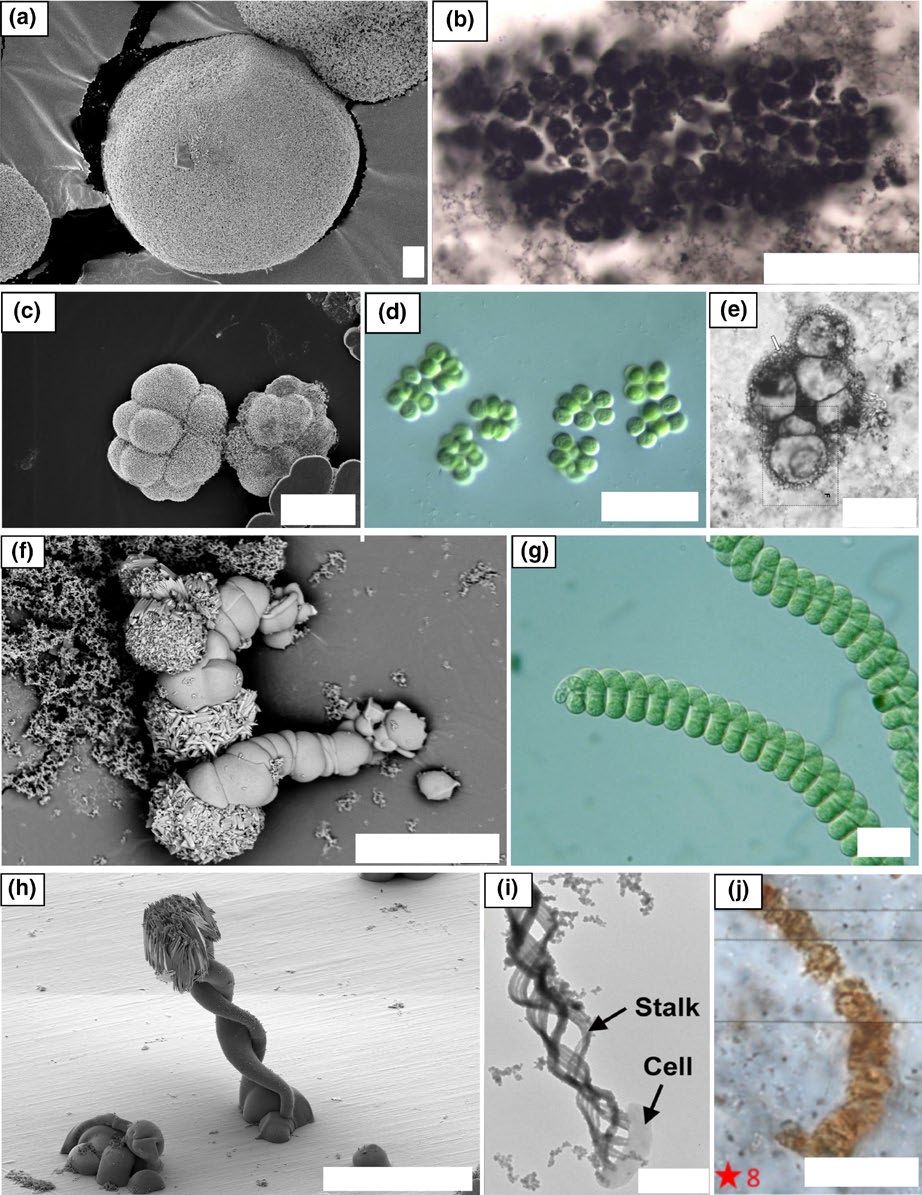
Morphological comparison of specific biomorph shapes, bacteria (or bacteria‐related structures) and Archean microstructures. (a) spheroidal biomorph, (b) population of spheroids from the Strelley Pool Formation, (c) framboidal biomorphs, (d) cluster of *Microcystis flosaquae,* (e) cluster of spheroids from the Strelley Pool formation, (f) wormlike biomorphs, (g) *Spirulina* sp. (h) helical braid‐like biomorph, (i) helical stalks produced by *Mariprofundus ferrooxydans*, (j) filamentous structures from Apex Chert. Sources of the pictures: (a), (c), (f), (h) this study. (b), (e) Sugitani et al. (2015). (d), (g) Pictures by Pr. Tsukii, Protist Information Server. (i) Singer et al. (2011). (j) Wacey et al. (2015). Scalebars: (a), (i) 1 μm; (b), (d), (e), (f), (g), (h) 50 μm; (c), (j) 10 μm [Colour figure can be viewed at http://wileyonlinelibrary.com]

Many of the diverse biomorph shapes that we obtained in our solution experiments resemble shapes of modern micro‐organisms. The fractal growth process gives rise to biomorphs resembling common morphogroups of bacteria. Purely spheroidal biomorphs (Figure 10a) can be compared to the shapes of monococci bacteria, whereas framboidal biomorphs (Figure 10c) resemble for instance bacterial clusters obtained through the random divisional pattern of streptococci (e.g., *Microcystis flosaquae* in Figure 10d). When the internal geometry of biomorphs is accessible, they may be distinguished by their internal continuity, while individual microbial cells in clusters are separated by membranes and walls. In the Archean rock record, spheroidal and clustered microstructures have been identified in the Strelley Pool formation, the Farrell Quartzite, and the Moodies Group (Figure 10b,e; Javaux, Marshall, & Bekker, 2010; Sugitani, Grey, Nagaoka, Mimura, & Walter, 2009; Sugitani et al., 2010). The curvilinear growth process generates biomorphs with helicoidal structures such as braided (Figure 10h) or cylindrical, segmented (wormlike) shapes (Figure 10f), which can be compared to, for example, the stalks produced by *Mariprofundus ferroxydans* (Figure 10i, Singer et al., 2011), or a wide range of filamentous bacteria (e.g., *Oscillatoria princeps*). In the first case, there is an obvious difference in size. In the latter case, biomorphs can usually be distinguished by their shorter length and coiled internal geometry, although cyanobacteria such as *Spirulina* sp. display the same internal geometry (Figure 10g). Helicoidal, and more precisely wormlike biomorphs, have been compared before to the controversial Apex Chert microstructures (Garcia‐Ruiz et al., 2003; Figure 10j). It is important to note that the flanged microstructures found in the Strelley Pool Formation and the Farrell Quartzite (Sugitani, Mimura, Nagaoka, Lepot, & Takeuchi, 2013; Sugitani et al., 2009, 2010) have no morphological equivalents among the biomorphs grown in this study. This confirms the often discussed difficulty of distinguishing actual microbial remnants from abiotic counterparts based on morphological criteria alone (Brasier et al., 2002; Buick, 1990; Garcia‐Ruiz et al.,^2003^; Schopf et al., 2002), especially in ancient metamorphosed rocks.

#### 4.2.2 Variation of morphologies in populations of solution‐grown biomorphs

In contrast to individual morphologies, the variation in shape within a population of microstructures could potentially serve as a criterion to discriminate between an abiotic and biologic origin. As reported in our results (end of section 4.2.1) and as is seen in Figure 9a, for a single constant synthesis condition, biomorphs with a range of smoothly varying morphologies can be observed. For example, at a given time in a single starting solution, biomorphs that were formed through fractal growth can range from dendritic shapes to multiglobular, biglobular, or even spheroidal shapes. This demonstrates the influence of local heterogeneities (such as supersaturation values or impurities) in solutions. Micro‐organisms from a single strain, however, exhibit very similar shapes, and a microbial assemblage of several strains displays a discontinuous spectrum of morphologies. This criterion can be applied to Archean microfossils, for example, carbonaceous microstructures described in the 3,4 Ga Strelley Pool Chert (Sugitani et al., 2013). The structures described therein have spheroidal shapes and sizes (~10 μm) similar to some biomorphs. However, their shape is also remarkably homogeneous across the population. Based on our experimental results, it can be concluded that it is highly unlikely that fractal biomorphs grown from solution could have produced such a homogeneous population of spheroids, which disproves the biomorph hypothesis in this case.

### 4.3 Size as a criterion to detect biomorphs in the fossil record

#### 4.3.1 Size of solution‐grown biomorphs

The size range (from a few μm to a few tens of μm) displayed by biomorphs grown in solutions in the upper‐pH domains, noted 2 and 3 in Figure 6, lies within the range of prokaryotic sizes. At lower pH values (domain noted 1 in Figure 6), fractal‐type biomorphs often grow to several hundreds of micrometers in diameter, which is one or two orders of magnitude larger than individual modern prokaryotes. Only very few strains of modern bacteria reach these sizes. However, many bacteria tend to associate in colonies, forming for example clusters or filaments, with sizes comparable to the largest biomorphs observed in domain 1. Overall, the absolute size ranges of biomorphs and bacteria (individual or colonial) are similar. Therefore, it is concluded that the absolute size of individual microstructures does not represent a robust criterion for biogenicity of microstructures in the Archean rock record.

#### 4.3.2 Distribution of sizes in populations of gel‐grown biomorphs

In contrast to sizes of individual biomorphs or micro‐organisms, the frequency distribution of sizes of biomorph/micro‐organism in a sample, a thin section or a picture, is a powerful descriptor of an assemblage. As automated size measurement protocols have been developed (using Elastic Light Scattering or Epifluorescence Microscopy data), the size distribution of single strain unicellular bacterial populations could be studied more effectively than before (Harvey & Marr, 1966; Katz et al., 2003; Uysal, 2001). According to these studies, bacteria as diverse as *Escherichia coli*,* Synechococcus* spp., *Pseudomonas aeruginosa*,* Staphylococcus aureus* or *Bacillus subtilis* all display unimodal, slightly positively skewed distributions. Using pictures of *Synechocystis* spp. (PCC 6803) cultures taken in optical microscopy (bright field or DIC), we also found this kind of distribution (Figure 8b). This is in accordance with early mathematical models which took into account a binary division, a Gaussian distribution of sizes of cells entering division, a Gaussian distribution of sizes of daughter‐cells, and a dependency of the growth rate on the cell volume (Koch, 1966). Therefore, most monospecific unicellular populations can be expected to display this kind of distribution. Natural populations are usually composed of several strains, and their size distributions are thus expected to be plurimodal. However, in the Precambrian micropaleontological record, unimodal distributions are also reported (Butterfield & Chandler, 1992; Sugitani et al., 2013; Wacey et al., 2011). This can be ascribed to three potential factors: (i) micropaleontologists often make size measurements on groups of microstructures that were separated before, based on their morphology; (ii) some groups of organisms can be selectively lost during diagenesis because they are more fragile than others, reducing the resulting diversity in the fossil assemblage; (iii) the plurimodal nature of a distribution can be difficult to detect when the size of the population is small and the distribution of single strains overlap each other.

The width of size distributions is quantitatively compared (see section 3.4) using the Average/Standard deviation ratios (hereafter noted *A*/*SD*). This ratio was found to be lower for silica‐witherite biomorphs and for abiotic spherulites from the Gwna group (Wacey et al., 2011) than for bacteria (0.8 for the spherulites, between 1.7 and 2.7 for biomorphs, 4.9 for bacteria). In Schopf, Kudryavtsev, Sugitani, and Walter (2010) and in Wacey et al. (2011), the authors already mentioned the large width of the size distribution of pseudofossils (abiotic spherulites from the Gwna group, but also hematite pseudofossils from the Lakhanda formation and kerogen pseudofossils from the Marra–Mamba formation). The width of the size distributions indicates differences in the genetic process. While daughter‐cells arise through a cell division process from a mother‐cell, biomorphs, single crystals and crystal aggregates grow through an accretionary process. Several of the Precambrian microfossil populations studied here (Table S1; Butterfield & Chandler, 1992; Wacey et al., 2011) display high *A*/*SD* values similar to the value of the Synechocystis population (between 4 and 5), which indicates that these populations may represent single strain microbial cells. In contrast, a population of spheroidal cells from the Gunflint formation (Wacey et al., 2011) and a population of spheroids from Agu Bay (Butterfield & Chandler, 1992) display lower *A*/*SD* values (between 2 and 3). As their biogenicity is not questioned, these wide distributions may be interpreted as cryptic plurimodal distributions, produced by the assemblage of several strains of bacteria. Alternatively, these wide distributions could be explained by degradational gradients (Knoll & Golubic, 1979).

### 4.4 Spatial gradients displayed by gel‐grown biomorphs

In silica gel experiments, the diffusion process results in a continuous change in size, in density and morphology of biomorphs (see picture 1 and section 3.4) observable on a short spatial scale, a type of spatial gradient which is not observed in biological assemblages. In the geological record, the presence of this kind of gradient within cherts would provide a strong indication that: (i) the structures are abiotic, and (ii) that the precursor of the deposit had a viscosity high enough to allow slow diffusion processes.

### 4.5 Biomorph growth conditions and Archean paleoenvironments

In the current study we have chosen to perform biomorph synthesis by classical atmospheric CO_2_ diffusion into an alkaline, silica‐rich, and Ba‐rich solution, and to study the full range of morphologies that are formed at variable starting pH. During the Archean, this set of conditions could have been present in shallow–marine hydrothermal vent systems or subaerial geothermal springs. In our experiments, fractal growth can occur over a wide range of conditions, at pH values between 9.5 and 11.3 and [Ba] between 1.25 and 40 mmol/L (see Figure 6), while the curvilinear growth of helicoidal biomorphs is restricted between pH 10.1 and 11.3, and [Ba] between 10 and 40 mmol/L. These barium concentrations are much higher than that of modern seawater ([Ba] = 0.15 μmol/kg, Herzig & Hannington, 2006), and modern hydrothermal fluids ([Ba] = 1.3–54 μmol/kg, (Charlou et al., 2005; Ludwig, Kelley, Butterfield, Nelson, & Früh‐Green, 2006; Von Damm, 1995). However, in Archean hydrothermal fluids that leached predominantly ultramafic crust, the barium concentration may have been higher. For instance, komatiites from the Barberton Greenstone Belt, South Africa contain 2–93 ppm barium (Parman, Shimizu, Grove, & Dann, 2003), while typical MORB basalts contain 0.5 ppm barium (Workman & Hart, 2005). The fact that many Archean hydrothermal deposits contain barite (Nijman et al., 1998; Philippot et al., 2007; Van Kranendonk, 2006) suggests that significant amounts of barium were available in these hydrothermal fluids but also shows that, at the time these deposits formed, barium sulfate rather than barium carbonate would precipitate. The solubility product of barium sulfate (*K*_sp_ = 1.08 10^−10^ at *T* = 25°C) is slightly smaller than that of barium carbonate (*K*_sp_ = 2.58 10^−9^ at *T* = 25°C; Lide, 1947). This difference in solubility product between the two phases is so small that the formation of either barite or witherite will largely depend on the partial pressure of CO_2_ (in the ocean, hydrothermal fluids, or pore fluids), and on pH, which controls the speciation of carbonic acid. An overall high CO_2_ pressure and local high pH will create higher supersaturation values for witherite than for barite. Furthermore, under the reducing conditions of the Hadean and early Archean, in many environments, the concentration of dissolved sulfate would be much lower than today, and witherite instead of barite would form when barium was available. Note that for strontium and calcium, the other alkaline‐earth elements that form biomorphs, the solubility product of strontianite (SrCO_3_, *K*_sp_ = 5.60 10^−10^ at 25°C) is almost three orders of magnitude lower than that of celestite (SrSO_4_, *K*_sp_ = 3.44 10^−7^ at 25°C) while the solubility product of aragonite (the CaCO_3_ phase forming biomorphs, *K*_sp_ = 6.0 10^−9^ at 25°C) is five order of magnitude lower than the one of gypsum (*K*_sp_ = 3.14 10^−5^ at 25°C; Lide, 1947). Therefore, in silica‐rich alkaline solutions, strontium should precipitate in the form of strontianite‐silica biomorphs and calcium should precipitate in the form of aragonite‐silica biomorphs. Our experiments allow for the first time to limit the geochemical conditions under which carbonate‐silica biomorphs could have formed in surface environments of the early Earth.

## 5 CONCLUSION

The experimental study of the silica‐witherite biomorph system at ambient conditions demonstrates the extensive range of biomimetic morphologies that can be formed in this simple and abiogenic chemical system with only subtle changes in environmental conditions. All the obtained morphologies can be interpreted as different steps of a morphogenetic continuum according to a model previously described (García‐Ruiz et al., 2009). The values of barium concentration and pH, which influences the speciation of dissolved CO_2_ and SiO_2_ and therefore the mineral saturation indexes, have a strong effect on the final morphologies obtained. They control the number and the size of biomorphs, control a transition from the fractal to the curvilinear growth regime, and affect the twisting during curvilinear growth.

Several paleoenvironmental inferences point to plausible scenarios for the formation of biomorphs during the Archean Eon, either in solution or in gel. However, this study demonstrates that the high barium concentrations and high pH values necessary for biomorph growth to occur in surface environments (influenced by CO_2_ diffusion), strongly restrict the number of settings where this growth could have occurred during the Archean. It remains to be determined what limits are imposed on biomorph growth under an extended range of conditions, especially when fluids are warmer (the solubility product of witherite is lowered, but that of silica is increased) or contain more CO_3_^2−^ (the pH of the system is buffered, enabling more time for slow nucleation processes).

Because of their close resemblance to biological shapes and processes—for example, cell division—finding the right criteria to distinguish biomorphs from actual microfossils is crucial for life detection protocols. Based on this study, we can list three criteria to discard the biomorph hypothesis when looking for remnants of prokaryotic life: (i) Biomorph populations display a wide, bell‐like distribution of sizes while single strain, unicellular bacterial populations have a narrower size distribution and assemblages of several strains display a plurimodal distribution, (ii) Biomorphs grown in gel‐like environments display a short‐scale continuous variation in size and density, that is very rarely seen in biological communities, and (iii) Biomorphs display a smooth variation of shapes under constant [Ba]‐pH conditions.

## CONFLICT OF INTEREST

The authors declare that there is no conflict of interest regarding the publication of this article.
