# Supplementary material for: A morphogram for silica‐witherite biomorphs and its application to microfossil identification in the early earth rock record
Source: Geobiology. 2018 Feb 27;16(3):279–96. doi: 10.1111/gbi.12278 (PMC5947568; doi:10.1111/gbi.12278)
Supplement: Supplementary file 1 [file GBI-16-279-s001.docx]

Source n Average +- Standard Deviation (μm) A / SD (no unit)

*Synechocystis* culture 496 2.95+-0.6 4.9

Strelley Pool spheroids^1^ 218 10.6+-2.8 3.8

Strelley Pool tubular sheaths^1^ 44 10.9+-2.42 4.5

Bitter Springs Spheroidal cells^1^ 519 9.3+-1.9 4.9

Agu bay spheroids^2^ 2292 10.6+-3.7 2.9

Gunflint spheroidal cells^1^ 805 5.3+-2.5 2.1

Biomorphs gel (L1) 9432 18+-6.7 2.7

Biomorphs gel (L2) 1093 37+-14.8 2.5

Biomorphs gel (L3) 464 42+-24.7 1.7

Gwna group spherulites^1^ 304 9.2+-11.5 0.8

Suppplementary material – Table 1: Quantitative comparison of the size distribution of a living community (*Synechocystis sp. )* with size distributions from the paleontological record (1: data from the supplementary material *in* Wacey *et al.*, 2011; 2: data from Butterfield & Chandler, 1992) and size distributions of biomorphs in gel. n: number of particles considered in the size distributions
